# Comparative Genomics and Phylogenetic Analyses of *Christia vespertilionis* and *Urariopsis brevissima* in the Tribe Desmodieae (Fabaceae: Papilionoideae) Based on Complete Chloroplast Genomes

**DOI:** 10.3390/plants9091116

**Published:** 2020-08-28

**Authors:** Xue-Li Zhao, Zhang-Ming Zhu

**Affiliations:** 1 College of Forestry, Southwest Forestry University, Kunming 650224, China; zhaoxueli@swfu.edu.cn; 2 School of Ecology and Environmental Science & Yunnan Key Laboratory for Plateau Mountain Ecology and Restoration of Degraded Environments, Yunnan University, Kunming 650500, China

**Keywords:** plastid genome, next-generation sequencing, genome features, phylogeny, *Uraria*, Fabaceae

## Abstract

Taxonomic and phylogenetic relationships of *Christia*, *Urariopsis*, *Uraria* and related genera within the tribe Desmodieae (Fabaceae: Papilionoideae) have long been controversial. Here, we report the complete chloroplast (cp) genomes of *Christia vespertilionis* and *Urariopsis brevissima* and perform comparative and phylogenetic analyses with *Uraria lagopodioides* and other relatives in the Desmodieae. The cp genomes of *C. vespertilionis* and *U. brevissima* are 149,656 and 149,930 bp long, with 128 unique genes (83 protein-coding genes, 37 tRNA genes and 8 rRNA genes), respectively. Comparative analyses revealed 95-129 simple sequence repeats (SSRs) and eleven highly variable regions (*trnK-rbcL*, *rbcL-atpB*, *ndhJ-trnF*, *trnL-trnT*, *psbD-rpoB*, *accD-cemA*, *petA-psbL*, *psbE-petL*, *rps11-rps19*, *ndhF-ccsA*, and *rps15-ycf1*) among six Desmodieae species. Phylogenetic analyses clearly resolved two subtribes (Desmodiinae and Lespedezinae) of Desmodieae as monophyletic, and the newly reported *C. vespertilionis* and *U. brevissima* clustered in subtribe Desmodiinae. A sister relationship of *C. vespertilionis* to *U. lagopodioides* was supported. Evidence was presented to support the treatment of *Urariopsis* as a distinct genus rather than in synonymy with *Uraria*. The results provide valuable information for further studies on species delimitation, phylogenetics, population genetics, and the evolutionary process of speciation in the Desmodieae.

## 1. Introduction

Fabaceae is the third largest family of angiosperms and comprises over 19,500 species in ca. 765 genera [1,2,3]. In a recent study, the family was divided into six subfamilies: Caesalpinioideae, Cercidoideae, Detarioideae, Dialioideae, Duparquetioideae, and Papilionoideae [3]. The tribe Desmodieae (Benth.) Hutchinson belongs to subfamily Papilionoideae and comprises ca. 530 species within 32 genera [4]. Fruit characters are of high diversity and taxonomic value in Desmodieae [5]. Within Desmodieae, three genera (*Christia* Moench, *Urariopsis* Schindler, and *Uraria* Desvaux) have specialized fruits with folded articles. The genus *Christia* includes ca. ten species distributed in tropical and subtropical Asia and Australasia, and the genus *Urariopsis* comprises two species distributed in Southeast Asia. The genus *Uraria* contains ca. 20 species found in the Old World tropics. These three genera are thought to have close affinities in having specialized fruits with folded articles, but they differ in having peculiar shapes, i.e., the arrangement of the loments in a zig-zag pattern (*Uraria* and *Christia*) or not (*Urariopsis*) and fruits wholly enclosed by the calyx at maturity (*Christia*) or not (*Uraria* and *Urariopsis*) [6]. Due to these overlapping morphological traits, taxonomic and evolutionary relationships among these three genera have been controversial [4,6,7,8]. Although previous phylogenetic analyses based on nr*ITS* and a few cpDNA markers have provided valuable information to understand phylogenetic relationships within tribe Desmodieae [5,9], phylogenetic relationships of these three genera are still unclear because of insufficient polymorphic sites and limited sampling.

Chloroplasts are photosynthetic organelles. Chloroplast (cp) genomes in plants are independent genomes encoding an array of proteins in relation to photosynthesis, biosynthesis of starch, fatty acids, pigments, and amino acids [10,11]. The typical angiosperm cp genome is a circular, double-stranded DNA molecule with a quadripartite structure composed of a large single copy region (LSC), a small single copy region (SSC), and a pair of inverted repeats (IRs) [12]. In contrast with nuclear genomes, cp genomes in Fabaceae are uniparentally inherited and largely conserved in gene content, organization, and structure [13,14]. Due to its stable uniparental inheritance, the cp genome can be of great value for discriminating between closely related species and understanding their evolutionary relationships [15,16,17]. With the development of next-generation sequencing (NGS), complete plastome sequencing has increased dramatically. In recent years, phylogenomic studies of cp genomes have provided new insights into subfamilial relationships and species diversification within Fabaceae [18,19,20]. In Desmodieae, cp genomes of 10 species representing seven genera have been published [20,21], but the cp genomes of *Christia* and *Urariopsis* species are still unknown.

In this study, we report the complete cp genomes of *Christia vespertilionis* (L. f.) Bakh. f. ex Meeuwen and *Urariopsis brevissima* Y.C. Yang and P.H. Huang by using Illumina sequencing technology. Comparative genomics and phylogenetic analyses were conducted to (1) explore their genomic features compared with *Uraria lagopodioides* (Linnaeus) Candolle and other relatives in Desmodieae, (2) examine their SSRs, repetitive sequences and codon usage patterns, (3) observe the variable regions among Desmodieae taxa, (4) determine phylogenetic position of *C. vespertilionis* and *U. brevissima* within the Desmodieae.

## 2. Results

### 2.1. Genome Structural Features and Gene Content

**Figure 1:**
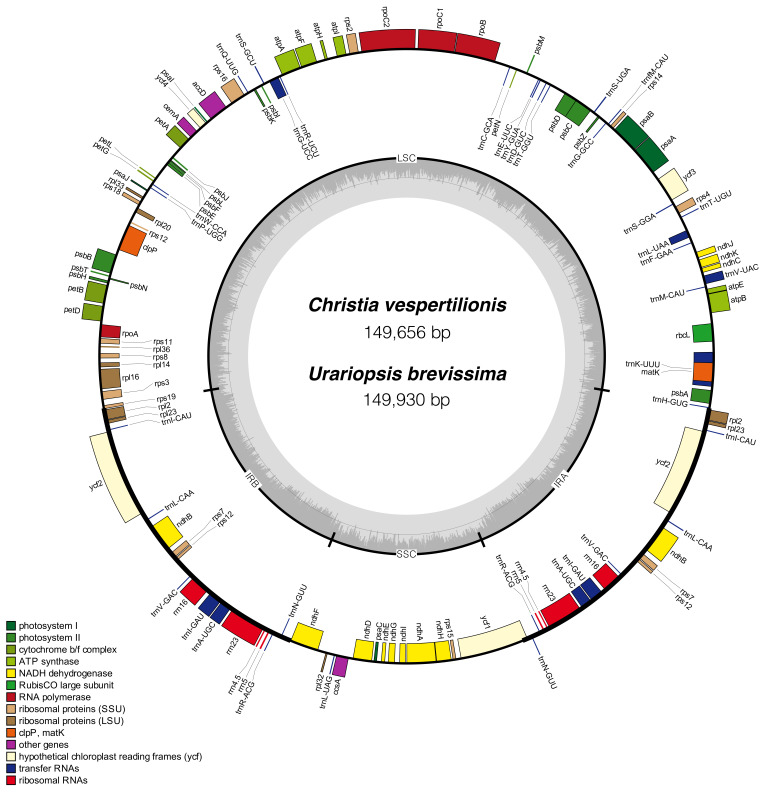
Gene maps of *Christia vespertilionis* and *Urariopsis brevissima* chloroplast genomes. Genes drawn outside the circle are transcribed counterclockwise, and those inside the circle are transcribed clockwise. Genes of different functions are color-coded. The dark gray area in the inner circle corresponds to the GC content of the corresponding genes, and the light gray area corresponds to the AT content.

The cp genomes of *C. vespertilionis* and *U. brevissima* are typical circular molecules with quadripartite structures containing a large single copy region (LSC) and a small single copy region (SSC) joined by two inverted repeats (IRA and IRB) (Figure 1). The sequences of *C. vespertilionis* and *U. brevissima* are 149,656 bp and 149,930 bp long, respectively. The LSC regions of *C. vespertilionis* and *U. brevissima* are 82,937 bp and 83,251 bp, respectively, and the sizes of their corresponding SSC regions are 18,451 bp and 18,403 bp, respectively. The basic features of these two cp genomes were compared to four other species in Desmodieae: *U. lagopodioides*, *Desmodium heterocarpon* (L.) DC., *Hylodesmum podocarpum* (DC.) H. Ohashi and R.R. Mill subsp. *podocarpum*, and *Ohwia caudata* (Thunberg) H. Ohashi (Table 1). The cp genomes of the six species ranged in size from 149,564 bp (*H. podocarpum* subsp. *podocarpum*) to 150,249 bp (*O. caudata*) (Table 1). Their overall GC contents were almost identical (35.2%), with the exception of 35.1% in *O. caudata*. However, the GC content in the four regions of each genome exhibited distinct differences. The lowest was observed in SSC (28.1%–28.5%), and the highest was observed in IR regions (42–42.1%) due to the presence of four rRNA genes (*rrn23*, *rrn16*, *rrn5*, and *rrn4.5*) in these regions.

Each cp genome included 128 genes: 83 protein-coding genes, 37 tRNA genes and eight rRNA genes (Table 1). In total, sixteen functional genes included five protein-coding genes (*ndhB*, *rpl12*, *rpl23*, *rps7*, and *ycf2*), four rRNA genes (*rrn23*, *rrn16*, *rrn5*, and *rrn4.5*) and seven tRNA genes (*trnA-UGC*, *trnI-CAU*, *trnI-GAU*, *trnL-CAA*, *trnN-GUU*, *trnR-ACG*, and *trnV-GAC*) which were doubled in the IR regions of each cp genome. Additionally, eleven intron-containing genes were observed in the six species, including three genes (*clpP*, *rps12*, and *ycf3*) that contain two introns, and eight genes (*atpF*, *petB*, *petD*, *ndhA*, *trnG-UCC*, *trnK-UUU*, *trnL-UAA*, and *trnV-UAC*) that contain a single intron (Table S1). The *rps12* gene was located with a single 5′ end in the LSC region and a repeated 3′ end in both of the IR regions.

**Figure 2:**
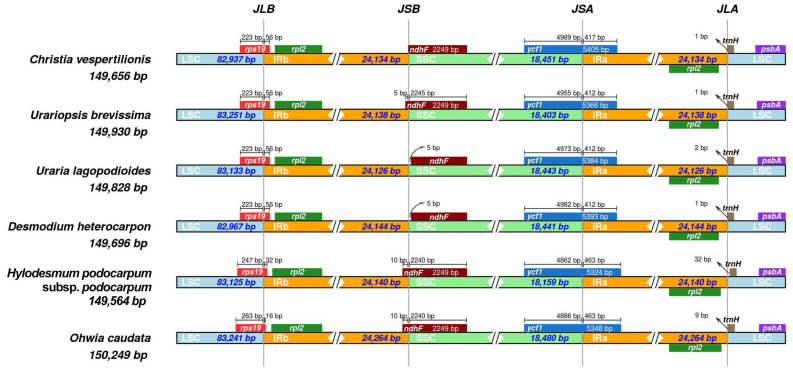
Comparison of the border position of LSC, IR and SSC among chloroplast genomes of six Desmodieae species.

Comparative analyses were performed among the six species for expansion and contraction in JLA (IRa-LSC), JLB (IRb-LSC), JSA (IRa-SSC), and JSB (IRb-SSC) (Figure 2). The IR regions range from 18,159 bp (*H. podocarpum* subsp. *podocarpum*) to 18,480 bp (*O. caudata*). The JLB, JSA, and JLA junctions are relatively conserved. In all six Desmodieae species, *rps19*, *ycf1*, and *trnH* are located in the JLB, JSA, and JLA junctions, respectively. Obvious variations were observed in the JSB junction, 5 bp (*U. brevissima*) to 10 bp (*H. podocarpum* subsp. *podocarpum* and *O. caudata*) of *ndhF* extended into the IR region, and no expansion or contraction of *ndhF* was detected in *C. vespertilionis*, while there was a 5 bp gap in *ndhF* away from the IR region in *U. lagopodioides* and *D. heterocarpon*.

### 2.2. Amino Acid Abundance and Codon Usage

**Figure 3:**
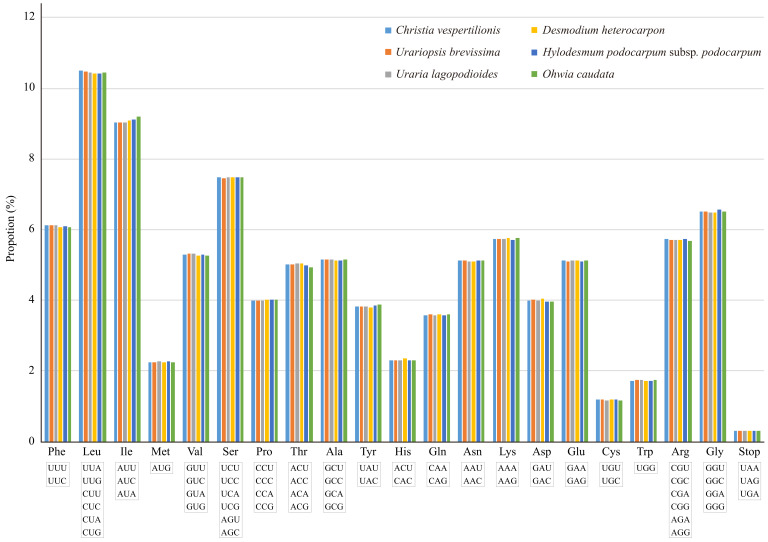
Amino acid frequencies in the protein-coding sequences of six Desmodieae species.

The 83 protein-coding genes (PCGs) are encoded by 26,021, 26,028, 26,010, 25,809, 25,975, and 26,015 codons in *C. vespertilionis*, *U. brevissima*, *U. lagopodioides*, *D. heterocarpon*, *H. podocarpum* subsp. *podocarpum*, and *O. caudata*, respectively (Table S2). Among these codons, 2691–2731 (10.43–10.50%) encode leucine, and 302–309 (1.16–1.20%) encode cysteine, the most and least abundant amino acids, respectively. The calculation of codon usage revealed that AAA (encoding lysine) was the most common synonymous codon, while UGC (encoding cysteine) was the least (Figure 3 and Figure S1; Table S2).

We calculated the relative synonymous codon usage (RSCU) to determine whether there is codon usage bias in coding sequences of these six species. Methionine AUG and tryptophan UGG had no usage bias (RSCU = 1). The highest RSCU value was for UUA (2.01 – 2.04) in leucine, indicating that the use of synonymous codons is more frequent than expected. The overall codon usage pattern in the six cp genomes tended toward A/U; among the 30 preferred codons (RSCU > 1), 29 ended with A/U.

### 2.3. Simple Sequence Repeats and Repetitive Sequence Analysis

**Figure 4:**
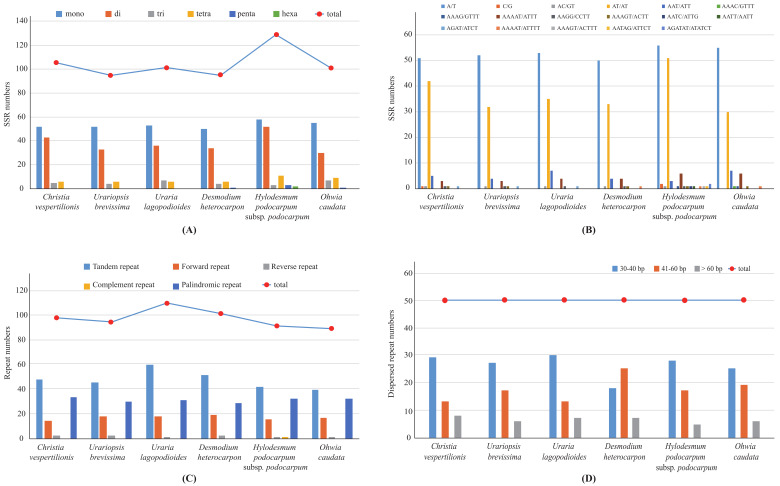
The number and type of simple sequence repeats (SSRs) and repeat sequences in the chloroplast genomes of six Desmodieae species. (**A**) Frequency of six SSR types, (**B**) Frequency of SSR motifs in different repeat class types, (**C**) Frequency of five repeat types, (**D**) Frequency of dispersed repeat sequences by length.

Ninety-five (*D. heterocarpon*) to 129 (*H. podocarpum* subsp. *podocarpum*) simple sequence repeats (SSRs) were identified in these six Desmodieae species (Figure 4A,B; Table S3). Mono-, di-, tri-, tetra-nucleotide SSRs were detected in all six species, but pentanucleotide and hexanucleotide were very rare. Most SSRs (48–55%) were mononucleotide A/T repeats, and only one and two C/G repeats were detected in *C. vespertilionis* and *H. podocarpum* subsp. *podocarpum*, respectively. The dinucleotide SSRs comprised 29–40% of all the detected SSRs, of which 97–100% were AT/AT repeats (Figure 4B). Although the richness of each type of SSR was similar in the six species, the number of SSRs differed among species, which can be used as molecular markers to identify them.

Our analyses revealed that the number of the five categories (tandem, forward, reverse, complement, and palindromic) of repeat sequences in the six Desmodieae cp genomes ranged from 90 (*H. podocarpum* subsp. *podocarpum*) to 110 (*U. lagopodioides*). Tandem repeats were the most common (44.44–54.55%), ranging from 40 (*O. caudata*) to 60 (*U. lagopodioides*), followed by palindromic repeats (28.18–35.56%), which ranged from 29 (*D. heterocarpon*) to 34 (*C. vespertilionis*). Only one pair of complement repeats was detected among all six species; it occurred in *H. podocarpum* subsp. *podocarpum* (Figure 4C; Table S4). All six species have the same total number (50) of dispersed repeats, of which repeats with a length of 30–40 bp were the most common, except in *D. heterocarpon* (Figure 4D).

### 2.4. Sequence Divergence Analysis

**Figure 5:**
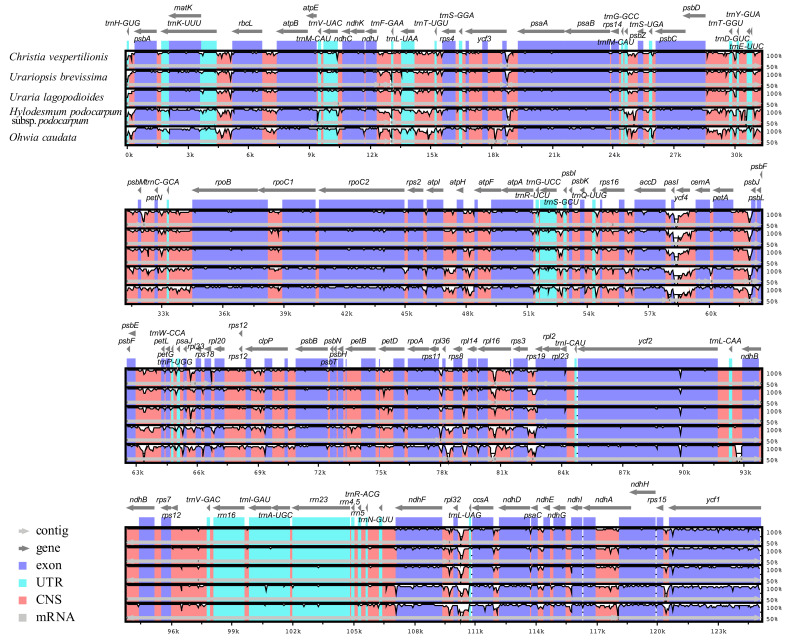
Alignment of the chloroplast genomes of six Desmodieae species, using *Desmodium heterocarpon* as a reference. The Y-scale represents the percent identity ranging from 50% to 100%.

**Figure 6:**
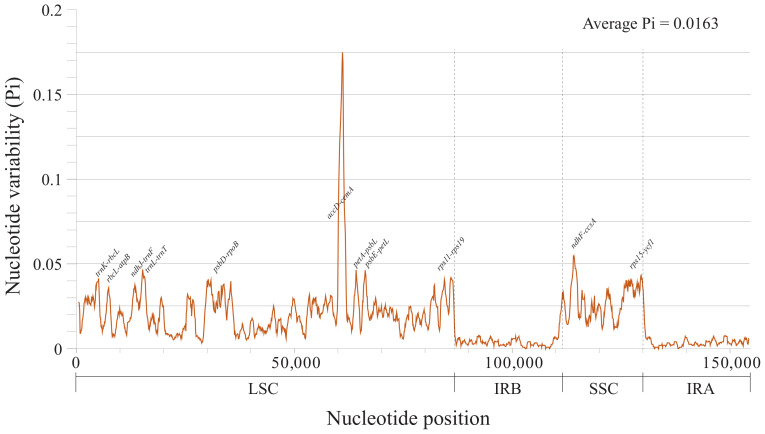
Nucleotide variability (Pi) values among chloroplast genomes of six Desmodieae species.

The annotated cp genome sequences of the six Desmodieae species were compared using mVISTA [22], with *D. heterocarpon* as a reference (Figure 5). The size and gene order of the cp genomes were conserved, but some divergent regions were identified, including ten intergenic regions (*trnK*-*rbcL*, *ndhJ*-*trnF*, *trnL*-*trnT*, *ycf3*-*psbA*, *trnG*-*trnS*, *trnQ*-*rps16*, *petA*-*psbJ*, *rps3*-*rps19*, *ndhF*-*rpl32*, and *rpl32*-*trnL*) and two coding regions (*ycf4* and *rpl16*) (Figure 5). Overall, higher divergence was detected in noncoding regions than in coding regions. In order to further assess the degree of sequence divergence, nucleotide variability (Pi) was calculated. The average value of Pi among the six Desmodieae species was estimated to be 0.0163, and the LSC and SSC regions were more divergent than the IR regions (Figure 6). Hotspot regions with Pi > 0.04 were identified: *trnK-rbcL*, *rbcL-atpB*, *ndhJ*-*trnF*, *trnL*-*trnT*, *psbD*-*rpoB*, *accD*-*cemA*, *petA*-*psbL*, *psbE*-*petL*, *rps11*-*rps19*, *ndhF*-*ccsA*, and *rps15*-*ycf1*. Among these, the *accD*-*cemA* region had a Pi value as high as 0.175 (Figure 6). These divergent regions would provide valuable information for marker development in the Desmodieae.

### 2.5. Phylogenetic Analysis

**Figure 7:**
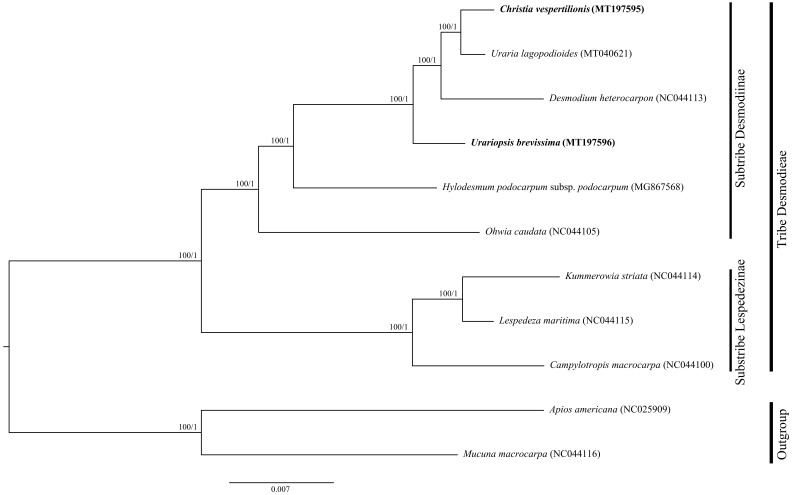
Phylogenetic tree inferred from maximum likelihood (ML) and Bayesian inference (BI) based on the whole chloroplast genomes. Numbers above the lines represent the ML bootstrap support values and Bayesian posterior probabilities, respectively.

Maximum likelihood (ML) and Bayesian inference (BI) trees based on the datasets comprising the complete cp genome sequences, LSC, SSC, IR, and PCGs showed identical phylogenetic topologies (Figure 7 and Figure S2). Phylogenetic analyses based on LSC, SSC, PCGs, and the complete cp genomes provided a better resolution of relationships (100% bootstrap support, 1.00 posterior probability in all the nodes) than did those based on IR region. The nine Desmodieae species clustered into two clades, reflecting subtribe Lespedezinae (Hutch.) Schubert and subtribe Desmodiinae Ohashi, Polhill et Schubert. *Christia vespertilionis* and *U. brevissima* clustered within subtribe Desmodiinae. All these phylogenetic trees supported a sister relationship of *C. vespertilionis* to *U. lagopodioides*. *Christia vespertilionis*, *U. lagopodioides*, and *D. heterocarpon* formed a well-supported monophyletic clade, which in turn formed a sister clade to *U. brevissima*.

## 3. Discussion

### 3.1. Comparative Analysis of Desmodieae Chloroplast Genomes

As in most angiosperms, cp genomes of six Desmodieae species are highly conserved in gene content, gene order, and intron number. However, expansion and contraction of the IR regions result in length variation of angiosperm cp genomes [23,24,25,26]. Generally, the length of IR regions differs by species. In this study, the length of the IR regions ranged from 18,159 bp to 18,480 bp. The largest IR region was found in the largest cp genome, that of *O. caudata* (150,249 bp), and the smallest IR region was found in the smallest cp genome, that of *H. podocarpum* subsp. *podocarpum* (149,564 bp). However, genome size varies in the expansion/contraction regions among different lineages, which can be used to conduct the phylogenetic studies [26].

SSRs in the cp genome (cpSSRs) often present high diversity in copy number and are important molecular markers for population genetics and evolutionary studies [27,28,29]. In this study, each cp genome was found to contain 95-129 SSRs, and the types of SSRs also differed by species. The SSRs of these cp genomes revealed abundant variation and can be used as molecular markers in population genetic studies of the Desmodieae, such as the genetic diversity analysis, speciation, and phylogeography of closely related species.

### 3.2. Phylogenetic Relationships of Desmodieae

Phylogenetic analyses based on relatively few genetic markers [5,9] have helped to elucidate phylogenetic relationships within the Desmodieae, but some intergeneric and interspecific relationships were poorly resolved due to insufficient polymorphic sites. Chloroplast genomes with sufficient informative sites can clarify difficult phylogenetic relationships and have been successfully used to resolve phylogenetic relationships at almost any taxonomic level [20,30,31]. In our study, phylogenetic trees inferred from different methods based on the complete cp genomes, LSC, SSC, IR, and PCGs showed an identical topology, with high resolution at each node, which indicates that these datasets taken collectively are useful for reconstructing phylogenetic relationships among the Desmodieae.

The two subtribes of Desmodieae (Desmodiinae and Lespedezinae) were supported as monophyletic clades in this study, which is consistent with the results of previous phylogenetic studies [5,13]. Consistent with traditional classifications, the newly reported *C. vespertilionis* and *U. brevissima* clustered in the subtribe Desmodiinae.

Based on morphological traits, *Christia* and *Uraria* are thought to have close affinities, both having specialized fruits with a zig-zag folded pattern. Our phylogenetic analyses provided robust support for the sister relationship of *C. vespertilionis* to *U. lagopodioides*, consistent with previous morphological, palynological, and molecular phylogenetic studies [5,9,32].

The systematic positions of *Urariopsis* have long been highly controversial since it was segregated from the genus *Uraria* by Schindler [33]. *Urariopsis* species morphologically resemble *Uraria* species in having fruits with folded article and similar calyces but differ in the arrangement of the loments. Some researchers supported Schindler’s taxonomic revision [6,34,35,36,37], while others contended that *Urariopsis* should be lumped with *Uraria* [4,7,8,38]. The genus *Urariopsis* comprises only two species, i.e., *Urariopsis cordifolia* (Wall.) Schindl. distributed in Southwest Asia and *Urariopsis brevissima* endemic to China. Neither of these *Urariopsis* species has ever been sampled in previous phylogenetic studies [5,9]. Our study provided new insights into the previously unknown phylogenetic position of *Urariopsis*, demonstrating that it is sister to a well-supported monophyletic clade composed of *D. heterocarpon*, *C. vespertilionis*, and *U. lagopodioides*. Unexpectedly, *U. lagopodioides* showed a relatively close relationship with *C. vespertilionis* and *D. heterocarpon* rather than *U. brevissima*. Thus, our results support the treatment of *Urariopsis* as a distinct genus. However, taxonomic and phylogenetic relationships among *Desmodium* taxa have long been confused [9]; thus, reliable phylogenetic relationships among *Uraria*, *Christia*, *Urariopsis*, and *Desmodium* deserve further study based upon broader sampling.

### 3.3. Identification of Highly Variable Regions

Highly variable regions of the cp genomes can not only be used for phylogenetic analysis and species discrimination but also provide crucial information to explore species divergence and population structure [39,40]. The extent and types of genetic polymorphisms in different regions of the cp genome vary. In most plants, the single copy regions (LSC and SSC regions) of the cp genome are more variable than that are the IR regions [16,41,42]. In our study, nucleotide diversity, as quantified by Pi values across the six Desmodieae cp genomes, for SSC and LSC was 0.02752 and 0.02146, respectively, both of which were significantly higher than the Pi value for the IR regions (0.00374).

The tribe Desmodieae is morphologically diverse and is known as one of the most difficult taxonomic groups in Fabaceae. Although many taxonomic studies [5,9] have been conducted, the taxonomic positions and phylogenetic relationships of species in Desmodieae are still poorly resolved. In this study, eleven noncoding regions (*trnK-rbcL*, *rbcL-atpB*, *ndhJ*-*trnF*, *trnL*-*trnT*, *psbD*-*rpoB*, *accD*-*cemA*, *petA*-*psbL*, *psbE*-*petL*, *rps11*-*rps19*, *ndhF*-*ccsA*, and *rps15*-*ycf1*) were highly variable, with high Pi values (Pi > 0.04). Among these regions, *ndhJ-trnF* and *trnT-trnL* have provided valuable information to reconstruct the phylogenetic relationships in the tribe Desmodieae [9]. However, the other highly variable regions have never been involved in the phylogenetic analyses for this tribe. They will presumably be suitable for resolving phylogenetic relationships and genetic structure at the species and population level in Desmodieae.

## 4. Materials and Methods

### 4.1. Material Sampling and DNA Extraction

Mature, healthy leaves of *C. vespertilionis* and *U. brevissima* were collected from Ledong, Hainan, China (18°43′26″ N, 108°54′09″ E) in March 2019 and Huidong, Guangdong, China (23°07′55″ N, 114°47′20″ E) in October 2018, respectively. Voucher specimens were deposited at College of Forestry, Southwest Forestry University. Total genomic DNA was extracted from silica-dried leaves with a modified CTAB method [43]. Nine published Fabaceae cp genomes were included in our analyses (Table S5).

### 4.2. Genome Sequencing, Assembly, and Annotation

Genome sequencing was performed on an Illumina Nova Seq 6000 platform at Annoroad Gene Technology (Beijing, China). For each species, approximately 10.0 Gb of raw data were generated with pair-end 150 bp read length. Quality control of the raw sequence reads was accomplished with fastp v0.20.0 [44]. High-quality reads filtered by fastp were first used to screen out the cp-like reads by BWA-MEM [45], using *U. lagopodioides* (MT040621), *Desmodium heterocarpon* (L.) DC. (NC044113), and *Glycine max* (L.) Merr. (NC007942) as references. Then, selected reads were assembled with NOVOPlasty [46] and SPAdes version 3.0 [47]. Assembled contigs were checked and modified by Geneious Prime v2020.0.4 (https://www.geneious.com) to generate and cyclize the cp genomes. Initial annotations were made by using PGA [48] and revised with Geneious Prime based on the three reference genomes. The tRNA genes were further verified with tRNAscan-SE program [49,50]. The annotated cp genome sequences of *C. vespertilionis* and *U. brevissima* were submitted to GenBank under accession numbers MT197595 and MT197596, respectively. Chloroplast genome maps were illustrated by applying OrganellarGenomeDRAW [51].

### 4.3. Codon Usage

Codon usage was determined for all protein-coding genes. The relative synonymous codon usage (RSCU) values and codon usage, which were used to reveal the characteristics of the variation in synonymous codon usage, were determined with MEGA-X [52].

### 4.4. Simple Sequence Repeats and Repetitive Sequence Analysis

Simple sequence repeats (SSRs) were identified by using MISA [53], with the minimal repeat numbers set to 10, 5, 4, 3, 3, and 3 for mono-, di-, tri-, tetra-, penta-, and hexanucleotide sequences, respectively. Dispersed (forward, reverse, palindrome, and complementary) repeats were determined by running the REPuter program [54] with a minimum repeat size of 30 bp and similarities of 90%. Tandem repeats were identified by running the web-based Tandem Repeats Finder (https://tandem.bu.edu/trf/trf.html), with alignment parameters being set to 2, 7, and 7 for matches, mismatches, and indels, respectively.

### 4.5. Genome Comparison and Divergent Hotspots Identification

The cp genome sequences were aligned with MAFFT v7 [55]. Then, the cp genomes of the six Desmodieae species were compared by using Shuffle-LAGAN mode in mVISTA [22]. Expansion or contraction of the IR region was investigated and visualized by IR scope [56]. To identify the rapidly evolving molecular markers, a sliding window analysis was conducted for nucleotide variability (Pi) in the whole cp genome by using DnaSp v5.10 [57]. The window length was set to 800 bp, with a 100 bp step size.

### 4.6. Phylogeneitc Analyses

Phylogenetic analyses were conducted for the two species reported in this study and nine previously reported species of Fabaceae (Table S5). *Mucuna macrocarpa* Wall. and *Apios americana* Medik. were used as outgroups. Phylogenetic analyses were performed based on the following five datasets: (1) the complete cp genome sequence, (2) large single copy region (LSC), (3) small single copy region (SSC), (4) inverted repeat region (IR), and (5) protein-coding genes (PCGs). The complete cp genomes were aligned with MAFFT v7 [56]. Phylogenetic relationships were reconstructed by using the maximum likelihood (ML) and Bayesian inference (BI) methods. For ML and BI analyses, the best substitution model was tested based on the Akaike information criterion (AIC) by ModelTest-NG [58]. The best-fitting model in the analysis was GTR+I+G. Maximum likelihood analysis was performed in RAxML v.8.2.4 [59] with 1000 rapid bootstrap analyses, followed by a search for the best-scoring tree in one single run. Bayesian inference was implemented in MrBayes v.3.2.6 [60]. Four Markov chain Monte Carlo (MCMC) simulations were run for 10,000,000 generations, starting from random trees and sampling one tree every 1000 generations. The first 2,500,000 generations (25%) were discarded as burn-in and a 50% majority-rule consensus tree was then computed.

## 5. Conclusions

The complete cp genomes of *C. vespertilionis* and *U. brevissima* are newly reported in our study, and *Urariopsis* species was sampled for the first time in the molecular phylogenetic studies. By comparing these cp genomes with those which have been published for Desmodieae, we obtained valuable genetic resources, including SSRs, repetitive sequences, codon usage, and highly variable loci. The cp genomes provide a better resolved phylogenetic framework for these studied species in the tribe Desmodieae than previous studies. Therefore, cp genomes will be helpful for taxonomic and evolutionary studies of the tribe Desmodieae.

## Figures and Tables

**Table 1:** Comparison of the chloroplast genome features of six Desmodieae species.

Genome Features	*Christia vespertilionis*	*Urariopsis brevissima*	*Uraria lagopodioides*	*Desmodium heterocarpon*	*Hylodesmum podocarpum* subsp. *podocarpum*	*Ohwia caudata*
Accession number	MT197595	MT197596	MT040621	NC044113	MG867568	NC044105
Genome size (bp)	149,656	149,930	149,828	149,696	149,564	150,249
LSC length (bp)	82,937	83,251	83,133	82,967	83,125	83,241
SSC length (bp)	18,451	18,403	18,443	18,441	18,159	18,480
IR length (bp)	24,134	24,138	24,126	24,144	24,140	24,264
PCGs total length (bp)	78,063	78,084	78,030	77,427	77,925	78,045
Total number of genes	128	128	128	128	128	128
Protein-coding genes	83	83	83	83	83	83
tRNA	37	37	37	37	37	37
rRNA	8	8	8	8	8	8
Overall GC%	35.2	35.2	35.2	35.2	35.2	35.1
GC% in LSC	32.8	32.7	32.7	32.7	32.6	32.6
GC% in SSC	28.2	28.2	28.1	28.1	28.5	28.3
GC% in IR	42.1	42.1	42.1	42.1	42.1	42
GC% in PCGs	36.2	36.1	36.2	36.1	36.1	36

## Supplementary Materials

The following are available online at https://www.mdpi.com/2223-7747/9/9/1116/s1: Figure S1: Codon usage for 20 amino acids and stop codons in all protein-coding genes; Figure S2: Phylogenetic relationships inferred from maximum likelihood (ML) and Bayesian inference (BI) based on partition datasets of eleven cp genomes. (A) LSC region, (B) SSC region, (C) IR region, (D) PCGs region. Numbers above the lines represent the ML bootstrap support values and Bayesian posterior probabilities, respectively; Table S1: List of genes in the cp genomes of six Desmodieae species; Table S2: Codon frequencies and relative synonymous codon usage (RSCU) values of the cp genomes of six Desmodieae species; Table S3: Types and numbers of SSRs motifs in six Desmodieae cp genomes; Table S4: The numbers of tandem, forward, reverse, complement, and palindromic repeats in six Desmodieae cp genomes; Table S5: GenBank accession numbers for taxa used in the comparative analysis and phylogenetic tree construction.
