# Supplementary material for: Comparative Genomics and Phylogenetic Analyses of Christia vespertilionis and Urariopsis brevissima in the Tribe Desmodieae (Fabaceae: Papilionoideae) Based on Complete Chloroplast Genomes
Source: Plants (Basel). 2020 Aug 28;9(9):1116. doi: 10.3390/plants9091116 (PMC7570174; doi:10.3390/plants9091116)
Supplement: Supplementary file 1 [file plants-09-01116-s001.zip › Supplementary files_revised_20200827/Figure S1.pdf]

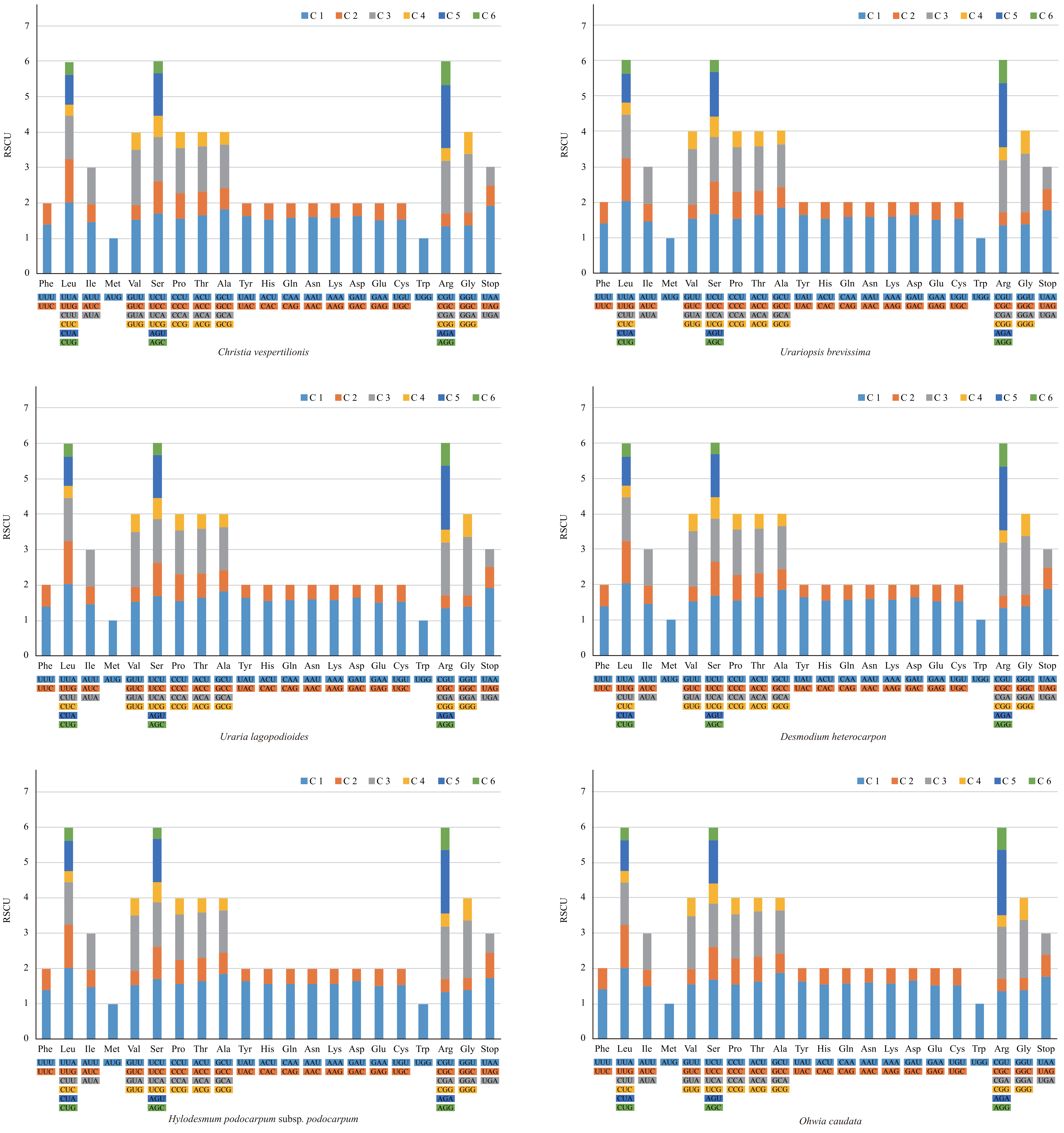
C 1 C 2 C 3 C 4 C 5 C 6
RSCU
0 1 2 3 4 5 6 7
Phe Leu Ile Met Val Ser Pro Thr Ala Tyr His Gln Asn Lys Asp Glu Cys Trp Arg Gly Stop
Phe: UUU UUC
Leu: UUA UUG CUU CUC CUA CUG
Ile: AUU AUC AUA
Met: AUG
Val: GUU GUC GUA GUG
Ser: UCU UCC UCA UCG AGU AGC
Pro: CCU CCC CCA CCG
Thr: ACU ACC ACA ACG
Ala: GCU GCC GCA GCG
Tyr: UAU UAC
His: ACU CAC
Gln: CAA CAG
Asn: AAU AAC
Lys: AAA AAG
Asp: GAU GAC
Glu: GAA GAG
Cys: UGU UGC
Trp: UGG
Arg: CGU CGC CGA CGG AGA AGG
Gly: GGU GGC GGA GGG
Stop: UAA UAG UGA
Christia vespertilionis
Urariopsis brevissima
Uraria lagopodioides
Desmodium heterocarpon
Hylodesmum podocarpum subsp. podocarpum
Ohwia caudata
