# Supplementary material for: Comparative Genomics and Phylogenetic Analyses of Christia vespertilionis and Urariopsis brevissima in the Tribe Desmodieae (Fabaceae: Papilionoideae) Based on Complete Chloroplast Genomes
Source: Plants (Basel). 2020 Aug 28;9(9):1116. doi: 10.3390/plants9091116 (PMC7570174; doi:10.3390/plants9091116)
Supplement: Supplementary file 1 [file plants-09-01116-s001.zip › Supplementary files_revised_20200827/Table S1.docx]

**Table S1** List of genes in the cp genomes of six Desmodieae species.

| Category for genes | Gene groups | Names of genes |
| --- | --- | --- |
| Photosynthesis related genes | Photosystem I | *psaA, psaB, psaC, psaI, psaJ* |
|  | Photosystem II | *psbA, psbB, psbC, psbD, psbE, psbF, psbH, psbI, psbJ, psbK, psbL,*  *psbM, psbN, psbT, psbZ* |
|  | ATP synthase | *atpA, atpB, atpE, atpF*^a^*, atpH, atpI* |
|  | Cytochrome b/f complex | *petA, petB*^a^*, petD*^a^*, petG, petL, petN* |
|  | Cytochrome c synthesis | *ccsA* |
|  | NADH-dehydrogenase | *ndhA*^a^*, ndhB(×2), ndhC, ndhD, ndhE, ndhF, ndhG, ndhH, ndhI, ndhJ, ndhK* |
|  | Rubisco | *rbcL* |
| Transcription and translation related genes | RNA polymerase | *rpoA, rpoB, rpoC1, rpoC2* |
|  | Proteins of small ribosomal subunit | *rps2, rps3, rps4, rps7(×2), rps8, rps11, rps12*^b^*(×2), rps14, rps15, rps16, rps18, rps19* |
|  | Proteins of large ribosomal subunit | *rpl2(×2), rpl14, rpl16, rpl20, rpl23(×2), rpl32, rpl33, rpl36* |
| RNA genes | Ribosomal RNAs | *rrn16(×2), rrn23(×2), rrn4.5(×2), rrn5(×2)* |
|  | Transfer RNAs | *trnA-UGC(×2), trnC-GCA, trnD-GUC, trnE-UUC, trnF-GAA, trnfM-CAU,*  *trnG-GCC, trnG-UCC*^a^*, trnH-GUG, trnI-CAU(×2), trnI-GAU(×2), trnK-UUU*^a^*, trnL-CAA(×2), trnL-UAA*^a^*, trnL-UAG, trnM-CAU,*  *trnN-GUU(×2), trnP-UGG, trnQ-UUG, trnR-ACG(×2), trnR-UCU,*  *trnS-GCU, trnS-GGA, trnS-UGA, trnT-GGU, trnT-UGU, trnV-GAC(×2),*  *trnV-UAC*^a^*, trnW-CCA, trnY-GUA* |
| Other genes | Carbon metabolism | *cemA* |
|  | Acetyl-CoA carboxylase | *accD* |
|  | Maturase | *matK* |
|  | Protease | *clpP*^b^ |
| Genes of unknown function | Conserved hypothetical chloroplast reading frames | *ycf1, ycf2(×2), ycf3*^b^*, ycf4* |

^a^Indicates the genes containing a single intron.

^b^Indicates the genes containing two introns.

(×2)Indicates genes duplicated in the IR regions.
