# Supplementary material for: Comparative Genomics and Phylogenetic Analyses of Christia vespertilionis and Urariopsis brevissima in the Tribe Desmodieae (Fabaceae: Papilionoideae) Based on Complete Chloroplast Genomes
Source: Plants (Basel). 2020 Aug 28;9(9):1116. doi: 10.3390/plants9091116 (PMC7570174; doi:10.3390/plants9091116)
Supplement: Supplementary file 1 [file plants-09-01116-s001.zip › Supplementary files_revised_20200827/Table S2.docx]

**Table S2** Codon frequencies and relative synonymous codon usage (RSCU) values of the cp genomes of six Desmodieae species.

| Species | Amino Acid | Codon | Count | RSCU | tRNA |
| --- | --- | --- | --- | --- | --- |
| *Christia vespertilionis* | Phe | UUU | 1112 | 1.4 |  |
|  |  | UUC | 482 | 0.6 | *trnF-GAA* |
|  | Leu | UUA | 922 | 2.03 | *trnL-UAA* |
|  |  | UUG | 548 | 1.21 | *trnL-CAA* |
|  |  | CUU | 554 | 1.22 |  |
|  |  | CUC | 150 | 0.33 |  |
|  |  | CUA | 378 | 0.83 | *trnL-UAG* |
|  |  | CUG | 169 | 0.37 |  |
|  | Ile | AUU | 1142 | 1.46 |  |
|  |  | AUC | 397 | 0.51 | *trnI-GAU* |
|  |  | AUA | 809 | 1.03 | *trnI-CAU* |
|  | Met | AUG | 589 | 1 | *trnfM-CAU, trnM-CAU* |
|  | Val | GUU | 530 | 1.53 |  |
|  |  | GUC | 146 | 0.42 | *trnV-GAC* |
|  |  | GUA | 536 | 1.55 | *trnV-UAC* |
|  |  | GUG | 174 | 0.5 |  |
|  | Ser | UCU | 543 | 1.68 |  |
|  |  | UCC | 305 | 0.94 | *trnS-GGA* |
|  |  | UCA | 403 | 1.24 | *trnS-UGA* |
|  |  | UCG | 192 | 0.59 |  |
|  | Pro | CCU | 402 | 1.55 |  |
|  |  | CCC | 194 | 0.75 |  |
|  |  | CCA | 324 | 1.25 | *trnP-UGG* |
|  |  | CCG | 117 | 0.45 |  |
|  | Thr | ACU | 539 | 1.64 |  |
|  |  | ACC | 222 | 0.68 | *trnT-GGU* |
|  |  | ACA | 415 | 1.27 | *trnT-UGU* |
|  |  | ACG | 136 | 0.41 |  |
|  | Ala | GCU | 611 | 1.82 |  |
|  |  | GCC | 199 | 0.59 |  |
|  |  | GCA | 410 | 1.22 | *trnA-UGC* |
|  |  | GCG | 120 | 0.36 |  |
|  | Tyr | UAU | 818 | 1.64 |  |
|  |  | UAC | 180 | 0.36 | *trnY-GUA* |
|  | His | CAU | 465 | 1.55 |  |
|  |  | CAC | 136 | 0.45 | *trnH-GUG* |
|  | Gln | CAA | 737 | 1.58 | *trnQ-UUG* |
|  |  | CAG | 195 | 0.42 |  |
|  | Asn | AAU | 1061 | 1.6 |  |
|  |  | AAC | 268 | 0.4 | *trnN-GUU* |
|  | Lys | AAA | 1174 | 1.57 | *trnK-UUU* |
|  |  | AAG | 317 | 0.43 |  |
|  | Asp | GAU | 849 | 1.64 |  |
|  |  | GAC | 189 | 0.36 | *trnD-GUC* |
|  | Glu | GAA | 1005 | 1.51 | *trnE-UUC* |
|  |  | GAG | 326 | 0.49 |  |
|  | Cys | UGU | 233 | 1.53 |  |
|  |  | UGC | 72 | 0.47 | *trnC-GCA* |
|  | Trp | UGG | 453 | 1 | *trnW-CCA* |
|  | Arg | CGU | 332 | 1.34 | *trnR-ACG* |
|  |  | CGC | 92 | 0.37 |  |
|  |  | CGA | 369 | 1.49 |  |
|  |  | CGG | 89 | 0.36 |  |
|  | Ser | AGU | 395 | 1.22 |  |
|  |  | AGC | 107 | 0.33 | *trnS-GCU* |
|  | Arg | AGA | 445 | 1.8 | *trnR-UCU* |
|  |  | AGG | 160 | 0.65 |  |
|  | Gly | GGU | 585 | 1.38 |  |
|  |  | GGC | 140 | 0.33 | *trnG-GCC* |
|  |  | GGA | 699 | 1.65 | *trnG-UCC* |
|  |  | GGG | 266 | 0.63 |  |
| *Urariopsis brevissima* | Phe | UUU | 1108 | 1.39 |  |
|  |  | UUC | 483 | 0.61 | *trnF-GAA* |
|  | Leu | UUA | 921 | 2.02 | *trnL-UAA* |
|  |  | UUG | 552 | 1.21 | *trnL-CAA* |
|  |  | CUU | 557 | 1.22 |  |
|  |  | CUC | 152 | 0.33 |  |
|  |  | CUA | 380 | 0.83 | *trnL-UAG* |
|  |  | CUG | 169 | 0.37 |  |
|  | Ile | AUU | 1144 | 1.46 |  |
|  |  | AUC | 396 | 0.51 | *trnI-GAU* |
|  |  | AUA | 809 | 1.03 | *trnI-CAU* |
|  | Met | AUG | 588 | 1 | *trnfM-CAU, trnM-CAU* |
|  | Val | GUU | 528 | 1.53 |  |
|  |  | GUC | 145 | 0.42 | *trnV-GAC* |
|  |  | GUA | 533 | 1.54 | *trnV-UAC* |
|  |  | GUG | 174 | 0.5 |  |
|  | Ser | UCU | 548 | 1.69 |  |
|  |  | UCC | 303 | 0.93 | *trnS-GGA* |
|  |  | UCA | 402 | 1.24 | *trnS-UGA* |
|  |  | UCG | 190 | 0.59 |  |
|  | Pro | CCU | 404 | 1.55 |  |
|  |  | CCC | 189 | 0.73 |  |
|  |  | CCA | 333 | 1.28 | *trnP-UGG* |
|  |  | CCG | 114 | 0.44 |  |
|  | Thr | ACU | 539 | 1.65 |  |
|  |  | ACC | 220 | 0.68 | *trnT-GGU* |
|  |  | ACA | 412 | 1.26 | *trnT-UGU* |
|  |  | ACG | 132 | 0.41 |  |
|  | Ala | GCU | 615 | 1.83 |  |
|  |  | GCC | 198 | 0.59 |  |
|  |  | GCA | 409 | 1.22 | *trnA-UGC* |
|  |  | GCG | 121 | 0.36 |  |
|  | Tyr | UAU | 818 | 1.64 |  |
|  |  | UAC | 181 | 0.36 | *trnY-GUA* |
|  | His | CAU | 464 | 1.54 |  |
|  |  | CAC | 137 | 0.46 | *trnH-GUG* |
|  | Gln | CAA | 730 | 1.57 | *trnQ-UUG* |
|  |  | CAG | 197 | 0.43 |  |
|  | Asn | AAU | 1063 | 1.6 |  |
|  |  | AAC | 268 | 0.4 | *trnN-GUU* |
|  | Lys | AAA | 1175 | 1.58 | *trnK-UUU* |
|  |  | AAG | 316 | 0.42 |  |
|  | Asp | GAU | 852 | 1.64 |  |
|  |  | GAC | 185 | 0.36 | *trnD-GUC* |
|  | Glu | GAA | 1010 | 1.51 | *trnE-UUC* |
|  |  | GAG | 326 | 0.49 |  |
|  | Cys | UGU | 235 | 1.53 |  |
|  |  | UGC | 73 | 0.47 | *trnC-GCA* |
|  | Trp | UGG | 451 | 1 | *trnW-CCA* |
|  | Arg | CGU | 331 | 1.33 | *trnR-ACG* |
|  |  | CGC | 95 | 0.38 |  |
|  |  | CGA | 366 | 1.47 |  |
|  |  | CGG | 90 | 0.36 |  |
|  | Ser | AGU | 397 | 1.22 |  |
|  |  | AGC | 108 | 0.33 | *trnS-GCU* |
|  | Arg | AGA | 445 | 1.79 | *trnR-UCU* |
|  |  | AGG | 163 | 0.66 |  |
|  | Gly | GGU | 581 | 1.37 |  |
|  |  | GGC | 147 | 0.35 | *trnG-GCC* |
|  |  | GGA | 698 | 1.65 | *trnG-UCC* |
|  |  | GGG | 268 | 0.63 |  |
| *Uraria lagopodioides* | Phe | UUU | 1114 | 1.4 |  |
|  |  | UUC | 479 | 0.6 | *trnF-GAA* |
|  | Leu | UUA | 926 | 2.04 | *trnL-UAA* |
|  |  | UUG | 545 | 1.2 | *trnL-CAA* |
|  |  | CUU | 559 | 1.23 |  |
|  |  | CUC | 149 | 0.33 |  |
|  |  | CUA | 377 | 0.83 | *trnL-UAG* |
|  |  | CUG | 168 | 0.37 |  |
|  | Ile | AUU | 1142 | 1.46 |  |
|  |  | AUC | 391 | 0.5 | *trnI-GAU* |
|  |  | AUA | 815 | 1.04 | *trnI-CAU* |
|  | Met | AUG | 584 | 1 | *trnfM-CAU, trnM-CAU* |
|  | Val | GUU | 528 | 1.53 |  |
|  |  | GUC | 142 | 0.41 | *trnV-GAC* |
|  |  | GUA | 535 | 1.55 | *trnV-UAC* |
|  |  | GUG | 177 | 0.51 |  |
|  | Ser | UCU | 540 | 1.67 |  |
|  |  | UCC | 299 | 0.92 | *trnS-GGA* |
|  |  | UCA | 402 | 1.24 | *trnS-UGA* |
|  |  | UCG | 191 | 0.59 |  |
|  | Pro | CCU | 403 | 1.55 |  |
|  |  | CCC | 194 | 0.74 |  |
|  |  | CCA | 330 | 1.27 | *trnP-UGG* |
|  |  | CCG | 115 | 0.44 |  |
|  | Thr | ACU | 539 | 1.65 |  |
|  |  | ACC | 219 | 0.67 | *trnT-GGU* |
|  |  | ACA | 408 | 1.25 | *trnT-UGU* |
|  |  | ACG | 141 | 0.43 |  |
|  | Ala | GCU | 616 | 1.84 |  |
|  |  | GCC | 193 | 0.58 |  |
|  |  | GCA | 410 | 1.22 | *trnA-UGC* |
|  |  | GCG | 123 | 0.37 |  |
|  | Tyr | UAU | 819 | 1.64 |  |
|  |  | UAC | 180 | 0.36 | *trnY-GUA* |
|  | His | CAU | 464 | 1.55 |  |
|  |  | CAC | 136 | 0.45 | *trnH-GUG* |
|  | Gln | CAA | 739 | 1.58 | *trnQ-UUG* |
|  |  | CAG | 199 | 0.42 |  |
|  | Asn | AAU | 1062 | 1.59 |  |
|  |  | AAC | 274 | 0.41 | *trnN-GUU* |
|  | Lys | AAA | 1181 | 1.58 | *trnK-UUU* |
|  |  | AAG | 313 | 0.42 |  |
|  | Asp | GAU | 856 | 1.64 |  |
|  |  | GAC | 190 | 0.36 | *trnD-GUC* |
|  | Glu | GAA | 1008 | 1.52 | *trnE-UUC* |
|  |  | GAG | 319 | 0.48 |  |
|  | Cys | UGU | 235 | 1.53 |  |
|  |  | UGC | 72 | 0.47 | *trnC-GCA* |
|  | Trp | UGG | 452 | 1 | *trnW-CCA* |
|  | Arg | CGU | 334 | 1.35 | *trnR-ACG* |
|  |  | CGC | 91 | 0.37 |  |
|  |  | CGA | 364 | 1.47 |  |
|  |  | CGG | 86 | 0.35 |  |
|  | Ser | AGU | 403 | 1.25 |  |
|  |  | AGC | 107 | 0.33 | *trnS-GCU* |
|  | Arg | AGA | 448 | 1.81 | *trnR-UCU* |
|  |  | AGG | 164 | 0.66 |  |
|  | Gly | GGU | 584 | 1.38 |  |
|  |  | GGC | 143 | 0.34 | *trnG-GCC* |
|  |  | GGA | 702 | 1.66 | *trnG-UCC* |
|  |  | GGG | 266 | 0.63 |  |
| *Desmodium heterocarpon* | Phe | UUU | 1090 | 1.39 |  |
|  |  | UUC | 477 | 0.61 | *trnF-GAA* |
|  | Leu | UUA | 917 | 2.04 | *trnL-UAA* |
|  |  | UUG | 533 | 1.19 | *trnL-CAA* |
|  |  | CUU | 553 | 1.23 |  |
|  |  | CUC | 148 | 0.33 |  |
|  |  | CUA | 372 | 0.83 | *trnL-UAG* |
|  |  | CUG | 168 | 0.37 |  |
|  | Ile | AUU | 1139 | 1.46 |  |
|  |  | AUC | 392 | 0.5 | *trnI-GAU* |
|  |  | AUA | 813 | 1.04 | *trnI-CAU* |
|  | Met | AUG | 580 | 1 | *trnfM-CAU, trnM-CAU* |
|  | Val | GUU | 519 | 1.52 |  |
|  |  | GUC | 143 | 0.42 | *trnV-GAC* |
|  |  | GUA | 530 | 1.56 | *trnV-UAC* |
|  |  | GUG | 170 | 0.5 |  |
|  | Ser | UCU | 543 | 1.69 |  |
|  |  | UCC | 305 | 0.95 | *trnS-GGA* |
|  |  | UCA | 394 | 1.23 | *trnS-UGA* |
|  |  | UCG | 190 | 0.59 |  |
|  | Pro | CCU | 401 | 1.55 |  |
|  |  | CCC | 189 | 0.73 |  |
|  |  | CCA | 331 | 1.28 | *trnP-UGG* |
|  |  | CCG | 115 | 0.44 |  |
|  | Thr | ACU | 535 | 1.64 |  |
|  |  | ACC | 218 | 0.67 | *trnT-GGU* |
|  |  | ACA | 409 | 1.26 | *trnT-UGU* |
|  |  | ACG | 139 | 0.43 |  |
|  | Ala | GCU | 611 | 1.84 |  |
|  |  | GCC | 196 | 0.59 |  |
|  |  | GCA | 401 | 1.21 | *trnA-UGC* |
|  |  | GCG | 117 | 0.35 |  |
|  | Tyr | UAU | 812 | 1.65 |  |
|  |  | UAC | 171 | 0.35 | *trnY-GUA* |
|  | His | CAU | 466 | 1.54 |  |
|  |  | CAC | 139 | 0.46 | *trnH-GUG* |
|  | Gln | CAA | 735 | 1.58 | *trnQ-UUG* |
|  |  | CAG | 198 | 0.42 |  |
|  | Asn | AAU | 1048 | 1.59 |  |
|  |  | AAC | 267 | 0.41 | *trnN-GUU* |
|  | Lys | AAA | 1178 | 1.58 | *trnK-UUU* |
|  |  | AAG | 311 | 0.42 |  |
|  | Asp | GAU | 859 | 1.65 |  |
|  |  | GAC | 183 | 0.35 | *trnD-GUC* |
|  | Glu | GAA | 998 | 1.51 | *trnE-UUC* |
|  |  | GAG | 323 | 0.49 |  |
|  | Cys | UGU | 236 | 1.53 |  |
|  |  | UGC | 73 | 0.47 | *trnC-GCA* |
|  | Trp | UGG | 446 | 1 | *trnW-CCA* |
|  | Arg | CGU | 330 | 1.34 | *trnR-ACG* |
|  |  | CGC | 87 | 0.35 |  |
|  |  | CGA | 368 | 1.5 |  |
|  |  | CGG | 86 | 0.35 |  |
|  | Ser | AGU | 394 | 1.23 |  |
|  |  | AGC | 102 | 0.32 | *trnS-GCU* |
|  | Arg | AGA | 443 | 1.8 | *trnR-UCU* |
|  |  | AGG | 159 | 0.65 |  |
|  | Gly | GGU | 574 | 1.37 |  |
|  |  | GGC | 141 | 0.34 | *trnG-GCC* |
|  |  | GGA | 699 | 1.67 | *trnG-UCC* |
|  |  | GGG | 263 | 0.63 |  |
| *Hylodesmum podocarpum* subsp. *podocarpum* | Phe | UUU | 1100 | 1.39 |  |
|  |  | UUC | 485 | 0.61 | *trnF-GAA* |
|  | Leu | UUA | 907 | 2.01 | *trnL-UAA* |
|  |  | UUG | 557 | 1.23 | *trnL-CAA* |
|  |  | CUU | 541 | 1.2 |  |
|  |  | CUC | 149 | 0.33 |  |
|  |  | CUA | 385 | 0.85 | *trnL-UAG* |
|  |  | CUG | 169 | 0.37 |  |
|  | Ile | AUU | 1162 | 1.47 |  |
|  |  | AUC | 386 | 0.49 | *trnI-GAU* |
|  |  | AUA | 819 | 1.04 | *trnI-CAU* |
|  | Met | AUG | 590 | 1 | *trnfM-CAU, trnM-CAU* |
|  | Val | GUU | 528 | 1.54 |  |
|  |  | GUC | 142 | 0.41 | *trnV-GAC* |
|  |  | GUA | 534 | 1.56 | *trnV-UAC* |
|  |  | GUG | 169 | 0.49 |  |
|  | Ser | UCU | 550 | 1.7 |  |
|  |  | UCC | 300 | 0.93 | *trnS-GGA* |
|  |  | UCA | 406 | 1.25 | *trnS-UGA* |
|  |  | UCG | 181 | 0.56 |  |
|  | Pro | CCU | 411 | 1.57 |  |
|  |  | CCC | 181 | 0.69 |  |
|  |  | CCA | 334 | 1.28 | *trnP-UGG* |
|  |  | CCG | 118 | 0.45 |  |
|  | Thr | ACU | 538 | 1.66 |  |
|  |  | ACC | 215 | 0.66 | *trnT-GGU* |
|  |  | ACA | 411 | 1.27 | *trnT-UGU* |
|  |  | ACG | 132 | 0.41 |  |
|  | Ala | GCU | 614 | 1.85 |  |
|  |  | GCC | 196 | 0.59 |  |
|  |  | GCA | 404 | 1.22 | *trnA-UGC* |
|  |  | GCG | 116 | 0.35 |  |
|  | Tyr | UAU | 820 | 1.64 |  |
|  |  | UAC | 183 | 0.36 | *trnY-GUA* |
|  | His | CAU | 468 | 1.57 |  |
|  |  | CAC | 130 | 0.43 | *trnH-GUG* |
|  | Gln | CAA | 731 | 1.57 | *trnQ-UUG* |
|  |  | CAG | 199 | 0.43 |  |
|  | Asn | AAU | 1054 | 1.58 |  |
|  |  | AAC | 279 | 0.42 | *trnN-GUU* |
|  | Lys | AAA | 1170 | 1.58 | *trnK-UUU* |
|  |  | AAG | 315 | 0.42 |  |
|  | Asp | GAU | 848 | 1.65 |  |
|  |  | GAC | 183 | 0.35 | *trnD-GUC* |
|  | Glu | GAA | 1005 | 1.52 | *trnE-UUC* |
|  |  | GAG | 318 | 0.48 |  |
|  | Cys | UGU | 237 | 1.54 |  |
|  |  | UGC | 71 | 0.46 | *trnC-GCA* |
|  | Trp | UGG | 450 | 1 | *trnW-CCA* |
|  | Arg | CGU | 332 | 1.34 | *trnR-ACG* |
|  |  | CGC | 93 | 0.38 |  |
|  |  | CGA | 366 | 1.48 |  |
|  |  | CGG | 89 | 0.36 |  |
|  | Ser | AGU | 398 | 1.23 |  |
|  |  | AGC | 109 | 0.34 | *trnS-GCU* |
|  | Arg | AGA | 449 | 1.81 | *trnR-UCU* |
|  |  | AGG | 158 | 0.64 |  |
|  | Gly | GGU | 596 | 1.4 |  |
|  |  | GGC | 141 | 0.33 | *trnG-GCC* |
|  |  | GGA | 696 | 1.63 | *trnG-UCC* |
|  |  | GGG | 274 | 0.64 |  |
| *Ohwia caudata* | Phe | UUU | 1109 | 1.4 |  |
|  |  | UUC | 470 | 0.6 | *trnF-GAA* |
|  | Leu | UUA | 913 | 2.02 | *trnL-UAA* |
|  |  | UUG | 546 | 1.21 | *trnL-CAA* |
|  |  | CUU | 549 | 1.21 |  |
|  |  | CUC | 151 | 0.33 |  |
|  |  | CUA | 393 | 0.87 | *trnL-UAG* |
|  |  | CUG | 163 | 0.36 |  |
|  | Ile | AUU | 1181 | 1.48 |  |
|  |  | AUC | 381 | 0.48 | *trnI-GAU* |
|  |  | AUA | 831 | 1.04 | *trnI-CAU* |
|  | Met | AUG | 586 | 1 | *trnfM-CAU, trnM-CAU* |
|  | Val | GUU | 529 | 1.55 |  |
|  |  | GUC | 147 | 0.43 | *trnV-GAC* |
|  |  | GUA | 517 | 1.51 | *trnV-UAC* |
|  |  | GUG | 175 | 0.51 |  |
|  | Ser | UCU | 547 | 1.69 |  |
|  |  | UCC | 295 | 0.91 | *trnS-GGA* |
|  |  | UCA | 398 | 1.23 | *trnS-UGA* |
|  |  | UCG | 189 | 0.58 |  |
|  | Pro | CCU | 408 | 1.56 |  |
|  |  | CCC | 188 | 0.72 |  |
|  |  | CCA | 328 | 1.26 | *trnP-UGG* |
|  |  | CCG | 121 | 0.46 |  |
|  | Thr | ACU | 520 | 1.62 |  |
|  |  | ACC | 227 | 0.71 | *trnT-GGU* |
|  |  | ACA | 414 | 1.29 | *trnT-UGU* |
|  |  | ACG | 125 | 0.39 |  |
|  | Ala | GCU | 627 | 1.87 |  |
|  |  | GCC | 186 | 0.56 |  |
|  |  | GCA | 406 | 1.21 | *trnA-UGC* |
|  |  | GCG | 120 | 0.36 |  |
|  | Tyr | UAU | 831 | 1.64 |  |
|  |  | UAC | 180 | 0.36 | *trnY-GUA* |
|  | His | CAU | 465 | 1.55 |  |
|  |  | CAC | 135 | 0.45 | *trnH-GUG* |
|  | Gln | CAA | 736 | 1.57 | *trnQ-UUG* |
|  |  | CAG | 201 | 0.43 |  |
|  | Asn | AAU | 1060 | 1.59 |  |
|  |  | AAC | 272 | 0.41 | *trnN-GUU* |
|  | Lys | AAA | 1177 | 1.57 | *trnK-UUU* |
|  |  | AAG | 321 | 0.43 |  |
|  | Asp | GAU | 848 | 1.65 |  |
|  |  | GAC | 183 | 0.35 | *trnD-GUC* |
|  | Glu | GAA | 1018 | 1.53 | *trnE-UUC* |
|  |  | GAG | 317 | 0.47 |  |
|  | Cys | UGU | 230 | 1.52 |  |
|  |  | UGC | 72 | 0.48 | *trnC-GCA* |
|  | Trp | UGG | 458 | 1 | *trnW-CCA* |
|  | Arg | CGU | 334 | 1.36 | *trnR-ACG* |
|  |  | CGC | 89 | 0.36 |  |
|  |  | CGA | 357 | 1.45 |  |
|  |  | CGG | 87 | 0.35 |  |
|  | Ser | AGU | 399 | 1.23 |  |
|  |  | AGC | 118 | 0.36 | *trnS-GCU* |
|  | Arg | AGA | 456 | 1.85 | *trnR-UCU* |
|  |  | AGG | 152 | 0.62 |  |
|  | Gly | GGU | 589 | 1.39 |  |
|  |  | GGC | 145 | 0.34 | *trnG-GCC* |
|  |  | GGA | 697 | 1.64 | *trnG-UCC* |
|  |  | GGG | 265 | 0.63 |  |
