# Supplementary material for: Comparative Genomics and Phylogenetic Analyses of Christia vespertilionis and Urariopsis brevissima in the Tribe Desmodieae (Fabaceae: Papilionoideae) Based on Complete Chloroplast Genomes
Source: Plants (Basel). 2020 Aug 28;9(9):1116. doi: 10.3390/plants9091116 (PMC7570174; doi:10.3390/plants9091116)
Supplement: Supplementary file 1 [file plants-09-01116-s001.zip › Supplementary files_revised_20200827/Table S3.docx]

**Table S3** Types and numbers of SSRs motifs in six Desmodieae cp genomes.

| Species | Mononucleotide | | Dinucleotide | | Trinucleotide | | Tetranucleotide | | Pentanucleotide | | Hexanucleotide | | Total |
| --- | --- | --- | --- | --- | --- | --- | --- | --- | --- | --- | --- | --- | --- |
|  | No. | % | No. | % | No. | % | No. | % | No. | % | No. | % | No. |
| *Christia vespertilionis* | 52 | 49.06 | 43 | 40.57 | 5 | 4.72 | 6 | 5.66 | 0 | 0 | 0 | 0 | 106 |
| *Urariopsis brevissima* | 52 | 54.74 | 33 | 34.74 | 4 | 4.21 | 6 | 6.32 | 0 | 0 | 0 | 0 | 95 |
| *Uraria lagopodioides* | 53 | 51.96 | 36 | 35.29 | 7 | 6.86 | 6 | 5.88 | 0 | 0 | 0 | 0 | 102 |
| *Desmodium heterocarpon* | 50 | 52.63 | 34 | 35.79 | 4 | 4.21 | 6 | 6.32 | 1 | 1.05 | 0 | 0 | 95 |
| *Hylodesmum podocarpum* subsp. *podocarpum* | 58 | 44.96 | 52 | 40.31 | 3 | 2.33 | 11 | 8.53 | 3 | 2.33 | 2 | 1.55 | 129 |
| *Ohwia caudata* | 55 | 53.92 | 30 | 29.41 | 7 | 6.86 | 9 | 8.82 | 1 | 0.98 | 0 | 0 | 102 |
