# Supplementary material for: Comparative Genomics and Phylogenetic Analyses of Christia vespertilionis and Urariopsis brevissima in the Tribe Desmodieae (Fabaceae: Papilionoideae) Based on Complete Chloroplast Genomes
Source: Plants (Basel). 2020 Aug 28;9(9):1116. doi: 10.3390/plants9091116 (PMC7570174; doi:10.3390/plants9091116)
Supplement: Supplementary file 1 [file plants-09-01116-s001.zip › Supplementary files_revised_20200827/Table S4.docx]

**Table S4** The numbers of tandem, forward, reverse, complement, and palindromic repeats in six Desmodieae cp genomes.

| Species | Tandem repeats | | Forward repeats | | Reverse repeats | | Complement repeats | | Palindromic repeats | | total |
| --- | --- | --- | --- | --- | --- | --- | --- | --- | --- | --- | --- |
|  | No. | % | No. | % | No. | % | No. | % | No. | % | No. |
| *Christia vespertilionis* | 48 | 48.98 | 14 | 14.29 | 2 | 2.04 | 0 | 0.00 | 34 | 34.69 | 98 |
| *Urariopsis brevissima* | 45 | 47.37 | 18 | 18.95 | 2 | 2.10 | 0 | 0.00 | 30 | 31.58 | 95 |
| *Uraria lagopodioides* | 60 | 54.55 | 18 | 16.36 | 1 | 0.91 | 0 | 0.00 | 31 | 28.18 | 110 |
| *Desmodium heterocarpon* | 52 | 50.98 | 19 | 18.63 | 2 | 1.96 | 0 | 0.00 | 29 | 28.43 | 102 |
| *Hylodesmum podocarpum* subsp. *podocarpum* | 42 | 45.65 | 16 | 17.39 | 1 | 1.09 | 1 | 1.09 | 32 | 34.78 | 92 |
| *Ohwia caudata* | 40 | 44.44 | 17 | 18.89 | 1 | 1.11 | 0 | 0.00 | 32 | 35.56 | 90 |
