# Supplementary material for: Comparative Genomics and Phylogenetic Analyses of Christia vespertilionis and Urariopsis brevissima in the Tribe Desmodieae (Fabaceae: Papilionoideae) Based on Complete Chloroplast Genomes
Source: Plants (Basel). 2020 Aug 28;9(9):1116. doi: 10.3390/plants9091116 (PMC7570174; doi:10.3390/plants9091116)
Supplement: Supplementary file 1 [file plants-09-01116-s001.zip › Supplementary files_revised_20200827/Table S5.docx]

**Table S5** GenBank accession numbers for taxa used in the comparative analysis and phylogenetic tree construction in this study.

| Species | GenBank accession number |
| --- | --- |
| *Christia vespertilionis* | MT197595 |
| *Urariopsis brevissima* | MT197596 |
| *Uraria lagopodioides* | MT040621 |
| *Desmodium heterocarpon* | NC044113 |
| *Hylodesmum podocarpum* subsp. *podocarpum* | MG867568 |
| *Ohwia caudata* | NC044105 |
| *Kummerowia striata* | NC044114 |
| *Lespedeza maritima* | NC044115 |
| *Campylotropis macrocarpa* | NC044100 |
| *Apios americana* | NC025909 |
| *Mucuna macrocarpa* | NC044116 |
