# Supplementary figures and images for: Comparative Genomics and Phylogenetic Analyses of Christia vespertilionis and Urariopsis brevissima in the Tribe Desmodieae (Fabaceae: Papilionoideae) Based on Complete Chloroplast Genomes
Source: Plants (Basel). 2020 Aug 28;9(9):1116. doi: 10.3390/plants9091116 (PMC7570174; doi:10.3390/plants9091116)

(A) LSC

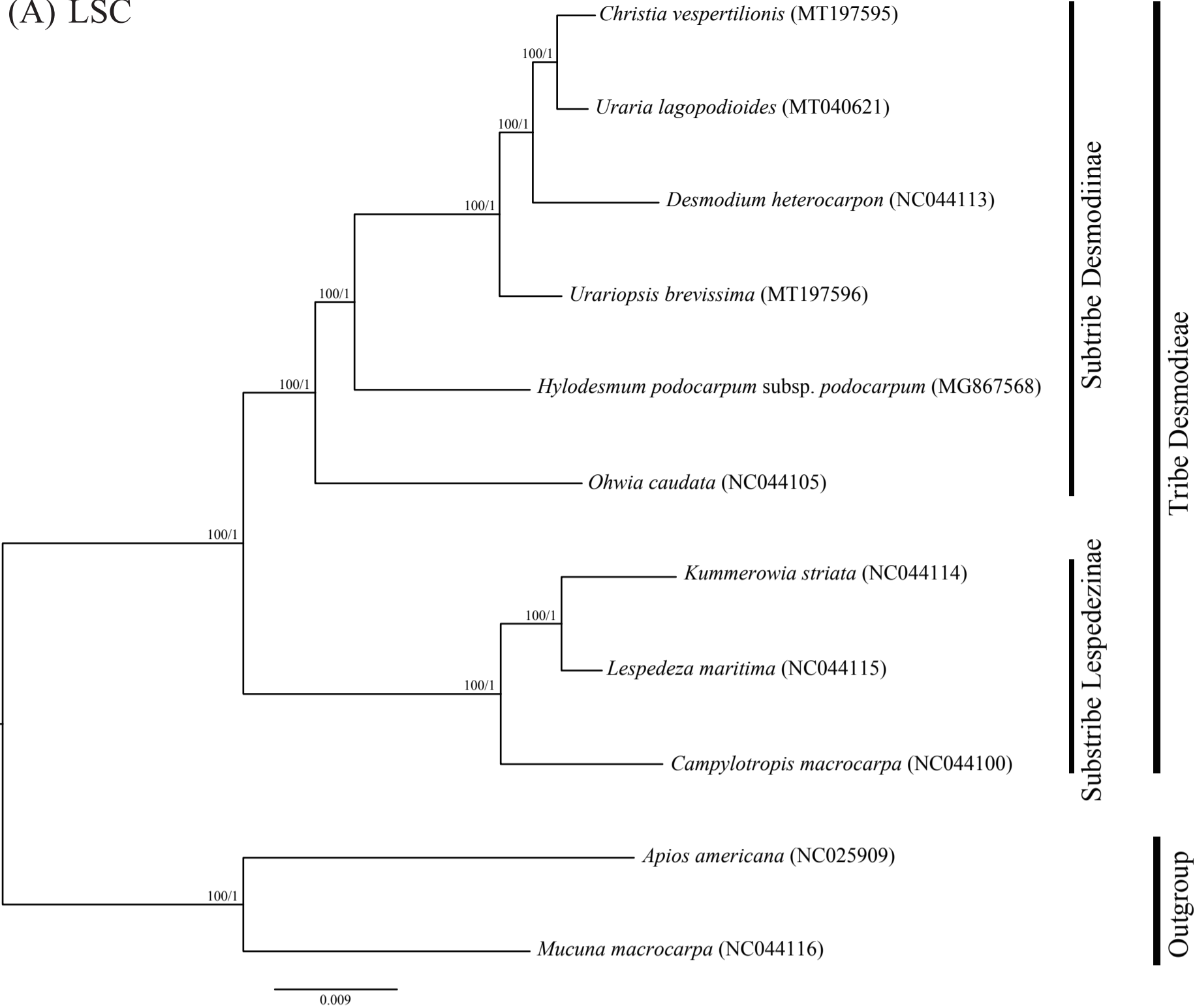

(B) SSC

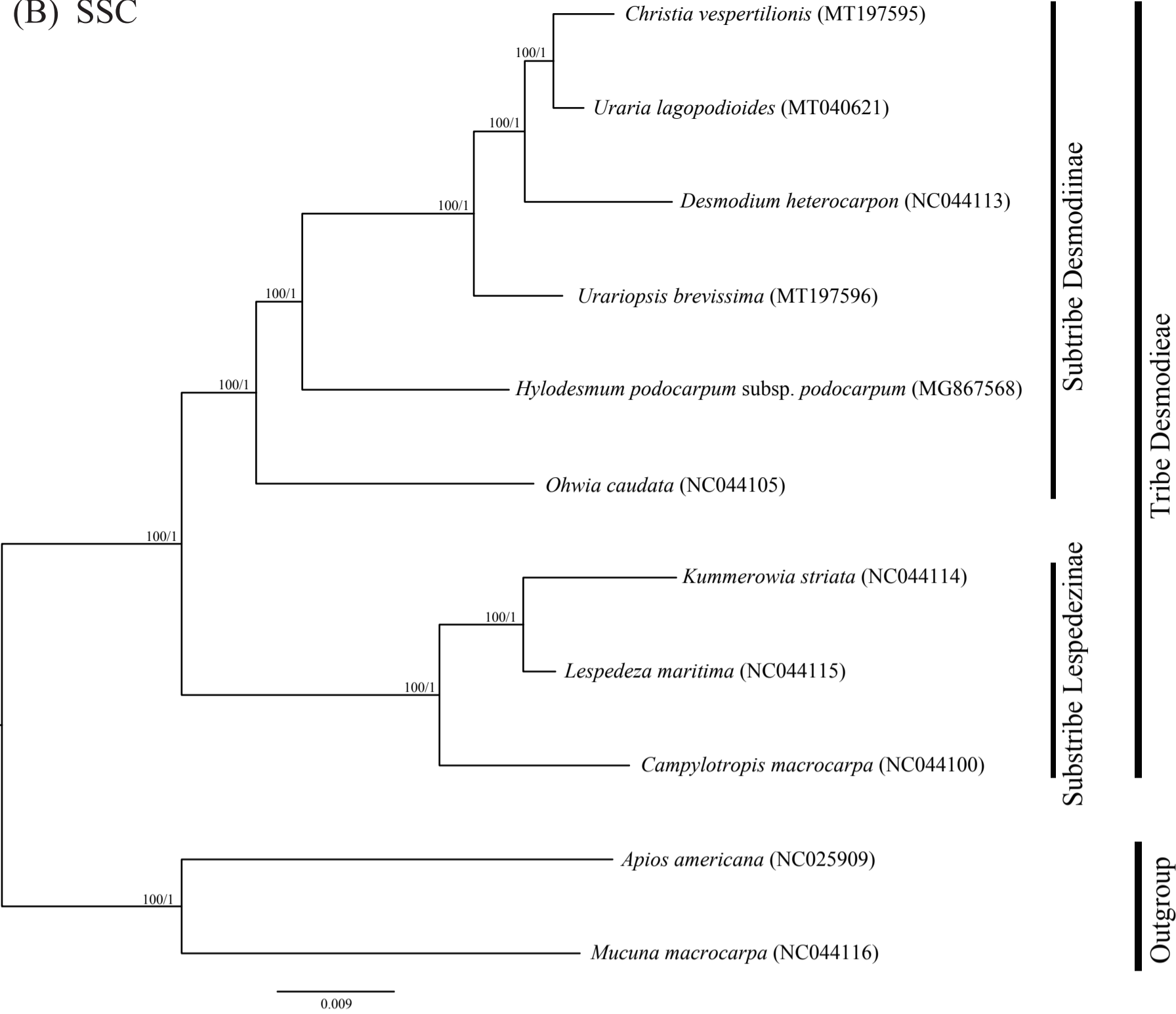

(C) IR

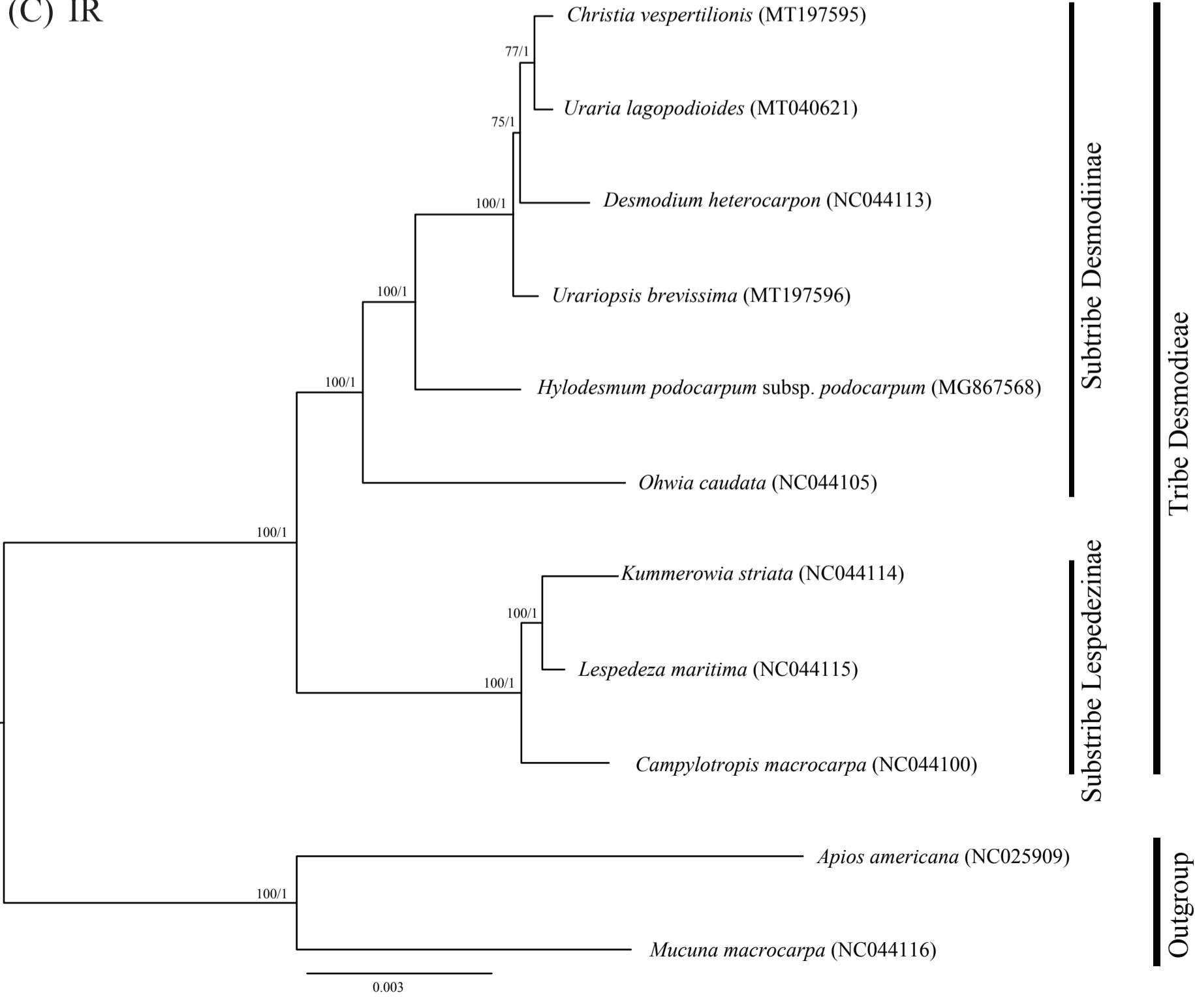

(D) PCGs

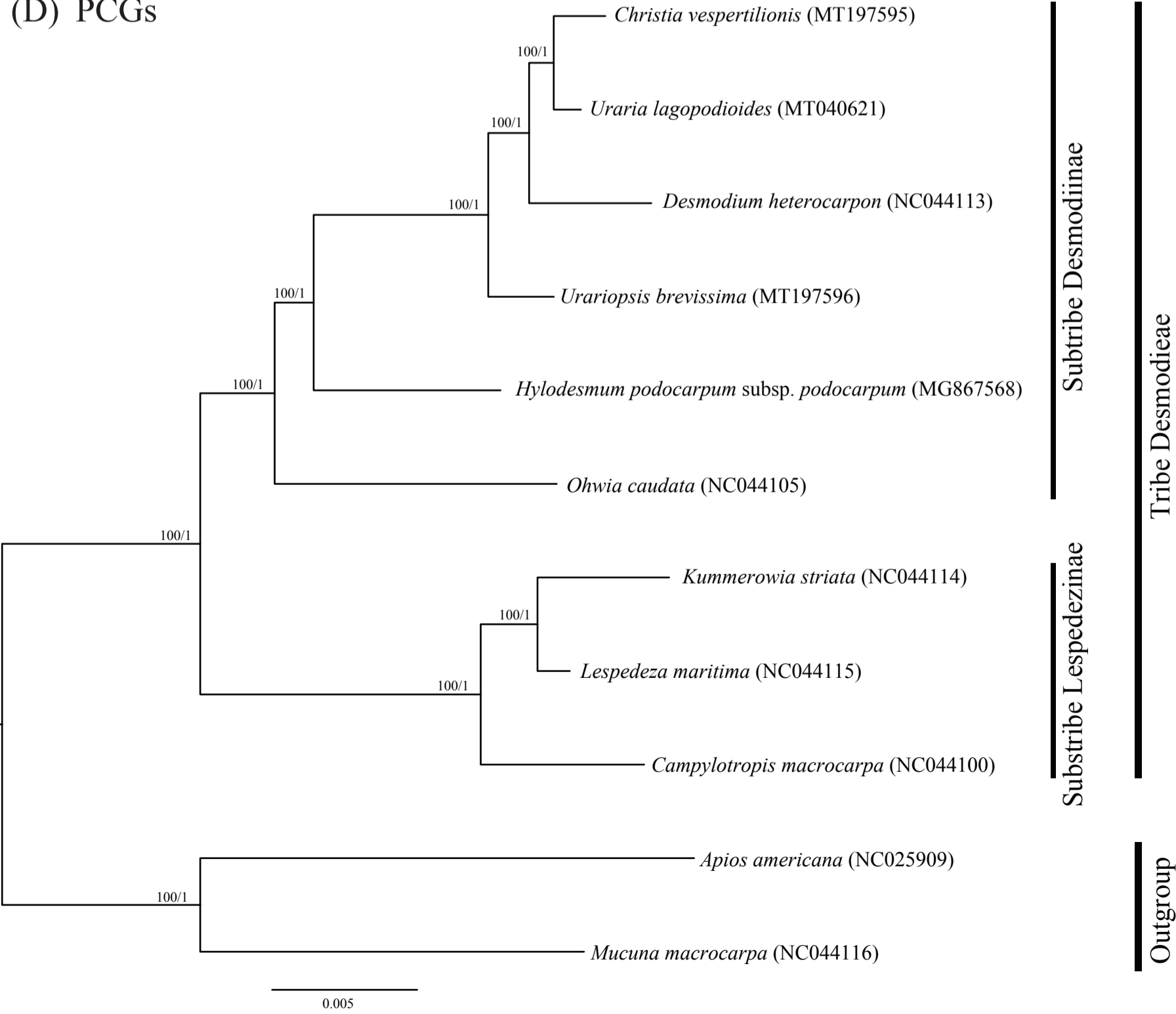

Supplement: Supplementary file 1 [file plants-09-01116-s001.zip › Supplementary files_revised_20200827/Figure S2.pdf]
